# Survival of entomopathogenic nematodes in oil emulsions and control effectiveness on adult engorged ticks (Acari: Ixodida)

**DOI:** 10.21307/jofnem-2019-001

**Published:** 2019-03-29

**Authors:** Teodulfo Aquino-Bolaños, Jaime Ruiz-Vega, Yolanda D. Ortiz Hernández, Julio C. Jiménez Castañeda

**Affiliations:** 1 Instituto Politécnico Nacional, Centro Interdisciplinario de Investigación para el Desarrollo Integral Regional Unidad Oaxaca, (CIIDIR-IPN-OAXACA), Hornos 1003 Colonia Nochebuena Santa Cruz Xoxocotlán, Oaxaca CP 71230, México

**Keywords:** average lethal doses concentrations, *H. bacteriophora*, *S. carpocapsae*, *S. websteri*

## Abstract

Although their control is based on chemical products, the infestations by ticks *(Ixodes scapularis* Say) are causing great losses and damages in the livestock production worldwide. In this study, the survival of the entomopathogenic nematodes *Heterorhabditis bacteriophora, Steinernema carpocapsae*, and Steinernema websteri in vegetal oil suspension at concentrations of 13% and 33% and their effectiveness to control ticks at concentrations of 50 ± 5 and 100 ± 10 nematodes in oil suspensions of *Cymbopogon citratus, Pelargonium* sp, *Juniperus virginiana, Rosa* sp, and *Mentha piperita* were evaluated in lab conditions. In field conditions, the Lethal Concentration (LC90) of *S. websteri* in oil suspensions of *J. virginiana* and *C. citratus* in dogs infested with ticks was evaluated. In the laboratory, it was found that an oil emulsion of *C. citratus* and *J. virginiana* at 13% maintained the survival of *S. carpocapsae, H. bacteriophora*, and *S. websteri* from 55% to 60% for a period of 96 hr. The combination of the *S. websteri* nematode with 50 or 100 nematodes in oil emulsions of *J. virginiana* at 33% presented a control effectiveness of 80–100% in adult ticks 24 hr post-application. In field, the LC90 of 119 juveniles of *S. websteri* in oil emulsions of *J. virginiana* at 33% on domestic dogs presented an accumulated and a control effectiveness of 89% after 96 hr post-application. The combined application of *J. virginiana* and *S. websteri* could be a good alternative for the control of ticks. It was observed that the time of contact and the type of vegetable oil were crucial factors to increase the effectiveness of control.

The ticks (Acari: Ixodidae) are widely distributed in the tropical, subtropical and warm areas and are able to parasite vertebrates. In animals, the ticks climb from their feet to the ears, neck and the perianal zone. In these places, the skin is thinner which makes their feeding easier. As many other parasites, they can spread easily throughout their hosts because they hold onto them during long periods and when they finish eating they then drop off the host and go to a new place. This is very important for the dynamic of the illnesses they transmit and the risk of incursion of new pathogens in areas where they were absent (Díaz et al., 2012). It is estimated that around one billion of bovine livestock are found in tropical and subtropical zones exposed to the infestations by ticks and/or illnesses transmitted by them, causing great losses in livestock production (Cruz et al., 2016).

In Mexico, the conventional method to control the ticks is using ixodicides, chemical products that are available in the national market. However, the risk of generating populations of ticks resistant to the ixodicides is higher if its use is not handled as indicated by the standards (Rodríguez-Vivas et al., 2014). Nowadays, there are very few studies on the biological control of ticks using entomopathogenic nematodes (Kocan et al., 1998).

Entomopathogenic nematodes (EPNs) are efficient agents of biological control, and are compatible with some chemical pesticides (Kaya and Gaugler, 1993). The effectiveness of the EPNs in tick's control depends on the time of permanency of the formulation on the pest's body because the infective juveniles (IJs) require enough time to find a host and need protection against adverse abiotic factors such as solar radiation and low moisture. Therefore, when the EPNs are applied in aqueous suspension the efficiency generally decreases. In this work, we aimed to identify the most beneficial combination of a vegetable oil emulsion to extend the shelf-life of EPNs at room temperature and to increase their infectivity on ticks in laboratory and field evaluations.

The compatibility of some vegetable oils with the nematode *Heterorhabditis bacteriophora* has been reported (Krishnayya and Grewal, 2002; Alves et al., 2017). Furthermore, owing to its viscosity level, the oil sticks for some time and its evaporation is slower compared with water. These physicochemical characteristics can be exploited in the application of EPN by delaying its desiccation and improving their interaction with the insect-pest.

Hence, in an effort to increase the survival time and infectivity of EPNs on ticks, this study aimed to evaluate: (i) the effect of five vegetable oil emulsions on the survival and infectivity of *S. carpocapsae*, *S. websteri* and *H. bacteriophora* on ticks in laboratory and (ii) the control effectiveness (CE) of application of EPNs in oil emulsion on ticks present in infested dogs (*Canis lupus familiaris*) in field conditions.

## Materials and methods

### Description of the area of study

This work was carried out under lab and field conditions. The lab experiments were done in the Laboratorio de Nematodos Entomopatógenos at CIIDIR-Oaxaca and the field experiments were done in Santa Cruz Xoxocotlán, Oaxaca, Mexico (17° 01′ 35″N, 96° 44′ 00″O, 1523 m altitude.

### Reproduction of entomopathogenic nematodes

The EPNs *Heterorhabditis bacteriophora* 18S and 28S HB1 Strain (Poinar, 1975), *Steinernema carpocapsae* 18S Access Gen Bank AF121049.1 (Weiser, 1955) were provided by the Department of Entomology of the University of California, Davis, USA, and *Steinernema websteri* 28SR and 28SF Access AY84172.1 (Cutler and Stock, 2003) is an native nematode that was isolated from soil in plantations of *Agave angustifolia* Haw by Delgado-Gamboa et al. (2015). The juveniles were reproduced on last instars larvae of the wax moth *Galleria mellonella*, following the method proposed by Kaya and Stock (1997). The temperature and relative humidity (RH) in laboratory were 19 to 25 °C and 37 to 64%, respectively. Three days after the inoculation, the infected larvae were set in White traps with filter paper discs (90 mm of diameter, Whatman No. 1). The emerging IJs were collected and maintained in distilled water in culture bottles of 250 mL and stored at 12 ± 1 °C for 24 hr before use.

### Collection of ticks

It was done by visual and tactile inspection of the presence of ticks in the infested dogs’ bodies. Adult ticks were collected by following the method proposed by Bonilla-Cárdenas et al. (2014), which consisted of holding the parasite with the index finger and thumb to separate it from the body of the animal pulling in a contrary direction of hair growth to avoid damages on it. In total, 60 adult ticks were collected and were kept in plastic containers of 100 mL which contained a piece of wet cotton to avoid dehydration of the organisms and to provide a suitable micro-environment to maintain their non-parasitic cycle. Furthermore, some 2 mm-holes were made in the lid for air exchange.

### Vegetable oils

Essential oils of Citronela (*Cymbopogon citratus* (de Candolle) Stapf) BIENAT^®^, Geranium (*Pelargonium* spp. Linnaeus) Nature's Bliss^®^, Juniper of virginia (*Juniperus virginiana* Linnaeus) ESENCIAL^®^, Roses (*Rosa* spp. Linnaeus) Nature's Bliss^®^, and Mint (*Mentha piperita* Linnaeus) BIENAT^®^ were used for the preparation of water-oil emulsions in different concentrations. These vegetable oils were purchased in local markets.

### Survival assays of entomopathogenic nematodes in emulsions

Two emulsions at two different vegetable oil concentrations (13 and 33%) were made. The amount of EPNs in each emulsion was 400 ± 20 IJs, but different amounts of oil and water were used (Table 1). These were placed in plastic petri dishes (55 mm of diameter), which were stored at room temperature (22 ± 3 °C and 37–64% RH). The survival of IJs in oil emulsions was assessed every 24 hr during 6 d. The observation and counting of living and dead IJs was done using a stereoscopic microscope. The IJs were considered alive if they had mobility in the head and tail by themselves (Peters, 2016).

**Table 1:** Treatments of EPNs in vegetable oil emulsions to evaluate the survival of IJs and control effectivity on adult ticks.

		Final composition		
Experiment	Treatment (%)	Oil (µL)	Amount of IJs	Distilled water (µL)
Survival	*Heterorhabdistis bacteriophora* – *C. citratus* 13	300	400 ± 20	2,000
	*Heterorhabditis bacteriophora*–*Pelargonium* 13	300	400 ± 20	2,000
	*Heterorhabditis bacteriophora* –*J. virginiana* 13	300	400 ± 20	2,000
	*Heterorhabditis bacteriophora* –*Rose* 13	300	400 ± 20	2,000
	*Heterorhabditis bacteriophora* –*M. piperita* 13	300	400 ± 20	2,000
	*Heterorhabditis bacteriophora* -AD (control)	0	400 ± 20	2,000
	*Steinernema carpocapsae* –*C. citratus* 13	300	400 ± 20	2,000
	*Steinernema carpocapsae* –*Pelargonium* 13	300	400 ± 20	2,000
	*Steinernema carpocapsae* –*J. virginiana* 13	300	400 ± 20	2,000
	*Steinernema carpocapsae* –*Rose* 13	300	400 ± 20	2,000
	*Steinernema carpocapsae* –*M. piperita* 13	300	400 ± 20	2,000
	*Steinernema carpocapsae* -AD (control)	0	400 ± 20	2,000
	*Steinernema websteri* –*C. citratus* 13	300	400 ± 20	2,000
	*Steinernema websteri* –*Pelargonium* 13	300	400 ± 20	2,000
	*Steinernema websteri* –*J. virginiana* 13	300	400 ± 20	2,000
	*Steinernema websteri* –*Rose* 13	300	400 ± 20	2,000
	*Steinernema websteri* –*M. piperita13*	300	400 ± 20	2,000
	*Steinernema websteri* -AD (Control)	0	400 ± 20	2,000
Control effectiveness in laboratory	*Steinernema websteri –J. virginiana* 33	833	50 ± 5	1,667
	*Steinernema websteri –C. citratus* 33	833	50 ± 5	1,667
	*Steinernema carpocapsae –J. virginiana* 33	833	50 ± 5	1,667
	*Steinernema carpocapsae –C. citratus* 33	833	50 ± 5	1,667
	*Heterorhabditis bacteriophora –J. virginiana* 33	833	50 ± 5	1,667
	*Heterorhabditis bacteriophora –C. citratus* 33	833	50 ± 5	1,667
	*Steinernema websteri –J. virginiana* 33	833	100 ± 10	1,667
	*Steinernema websteri –C. citratus* 33	833	100 ± 10	1,667
	*Steinernema carpocapsae –J. virginiana* 33	833	100 ± 10	1,667
	*Steinernema carpocapsae –C. citratus* 33	833	100 ± 10	1,667
	*Heterorhabditis bacteriophora –J. virginiana* 33	833	100 ± 10	1,667
	*Heterorhabditis bacteriophora –C. citratus* 33	833	100 ± 10	1,667
Control effectiveness in field	*Steinernema websteri –C. citratus* 33	833	119 ± 10	1,667
	*Steinernema websteri –J. virginiana* 33	833	119 ± 10	1,667
	*Steinernema websteri* -AD (Control)	0	119 ± 10	2,500

### Control effectiveness on ticks under laboratory conditions

The CE effectiveness of *S. websteri*, *S. carpocapsae* and *H. bacteriophora* nematodes in oil emulsions of *C. citratus* and *J. virginiana,* both at 33% of concentration, was assessed. The concentrations of EPNs were 50 ± 5 and 100 ± 10 IJs/tick. The ectoparasites were set individually in petri dishes with filter paper. The inoculation was performed after 12 hr with the oil emulsion of EPNs. The conditions of the ticks were registered every 24 hr until accumulating 100% of mortality; 120 adult ticks were used for this study. The cadavers were collocated in White traps to determine the cause of the death throughout necropsy tests, verifying if there were any EPNs, and the Koch's postulates were performed (Lacey and Solter, 2012).

### Control effectiveness on ticks in *C. lupus*

The EPNs were applied in oil emulsions of *J. virginiana* and *C. citratus* at 33% concentration in selected areas of infested domestic dogs, following the Mexican Standard NOM-006-ZOO (1993). The dogs were examined to identify and count the ticks. In total, 10 dogs with an individual weight of about 55 kg that were infested by ticks were selected, and four to six ticks per zone (feet, chest, cross, rump, muscular back and ears) were located for the bioassay. A total of 10 infested dogs with ticks with application of an aqueous suspension containing *S. websteri* were established as a control.

The application of the oil emulsions with EPNs on the infested areas was done in one single occasion. A disposable plastic syringe (BD Plastipak^®^ of 5 mL) was used to apply the treatments on the infested areas so that the emulsion could penetrate the dogs’ fur and skin. The control was treated with the same concentration of EPNs, but without vegetable oil. Evaluations were performed to all the fallen ticks or to the ones that presented signs of infection by EPNs, and the mortality of the ectoparasites was registered every 24 hr for 4 d. To verify infection by EPNs, each cadaver was washed twice with distilled water, transferred to White traps and incubated at 22 ± 3 °C until the IJs emerged.

### Experimental design

The experiments of survival of EPNs, the CE on adult ticks and the final composition of the evaluated treatments in the lab were done by duplicate with five repetitions per treatment. The field experiment on infested dogs with engorged adult ticks was done in a single occasion (Table 1).

### Statistical analysis

To determine the mortality of ticks, percentage data were transformed with the arcsine function. Then, variance analysis was carried out to establish the differences between the means by the Tukey test (*P* < 0.05). The LC_90_ was determined by Probit analysis of the data from the lab bioassay, which was used for the application of the EPNs in the effectiveness trials in the field. All the analyses were done in the statistic software SAS^®^ (Version 9.4; SAS Institute Inc., Cary, NC, USA).

## Results

### Survival of entomopathogenic nematodes in vegetable oil emulsions

**Figure 1:**
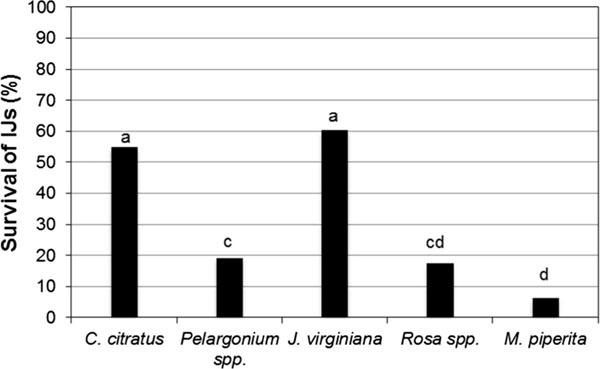
Survival of IJs in five vegetable oil emulsions at concentration of 13%.

The survival of *S. websteri, S. carpocapsae*, and *H. bacteriophora* was heterogeneous among the different oil emulsions because there was a wide variation from 6.14 to 60%. The lowest survival was obtained with the *M. piperita* oil emulsion and *Rose* oil emulsion. The most favorable emulsions for high survival percentages of IJs were the ones prepared with *J. virginiana* and *C. citratus,* the first one with a 60% and the second one with 55% survivorship, but without statistical differences between them (*P* > 0.05). However, they were statistically different (*α* < 0.005) from the emulsions of *Pelargonium*, *Rose*, and *M. piperita* (Fig. 1).

**Figure 2:**
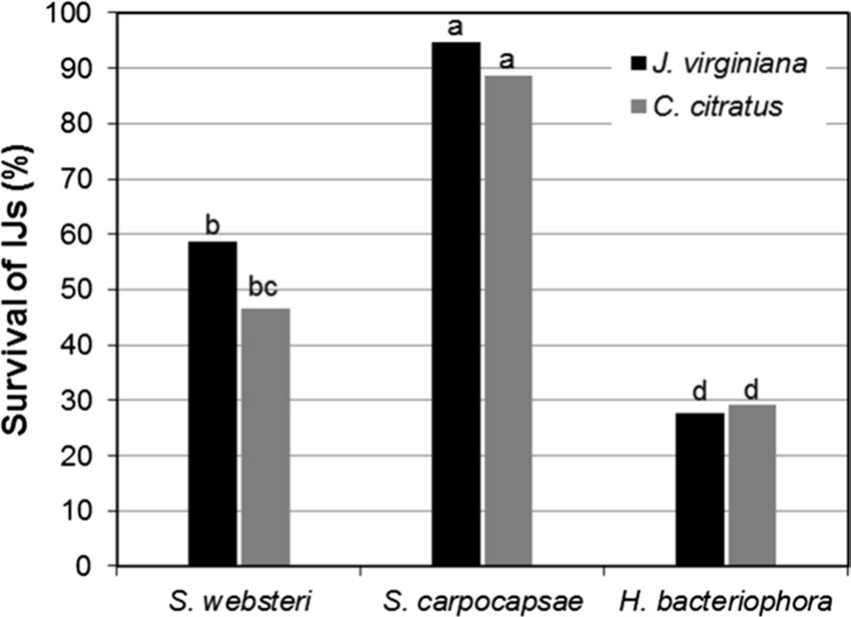
Survival of EPNs in oil emulsions of *J. virginiana* and *C. citratus* at a concentration of 13% under laboratory conditions.

It was observed that, because of desiccation, the emulsions began to lose mass and they hardened 72 hr after elaborated. Based on the survival of IJs, the data suggest that the vegetable oils of *C. citratus* and *J. virginiana* were the ones with the highest compatibility with the EPNs. The survival percentages of the nematodes *S. websteri, H. bacteriophora*, and *S. carpocapsae* in the oil emulsions of *C. citratus* and *J. virginiana* were the highest ones (Fig. 2).

*S. carpocapsae* 400 had the highest survival percentage in *J. virginiana* oil emulsion and *C. citratus*, with 95% and 88.7%, respectively. The second best treatment was the combination nematode *S. websteri* 400 in *J. virginiana* oil emulsions or *C. citratus* oil, with a variation of 46.66 to 58.80%. The combination with the lowest IJs survival percentage was *H. bacteriophora* in *J. virginiana* or *C. citratus* oil emulsions. The treatment with *H. bacteriophora* was statistically different (*P* = 0.05) from the treatments with the nematodes *S. websteri* and *S. carpocapsae*.

### Mortality of engorged adult ticks treated with EPN in laboratory

**Figure 3:**
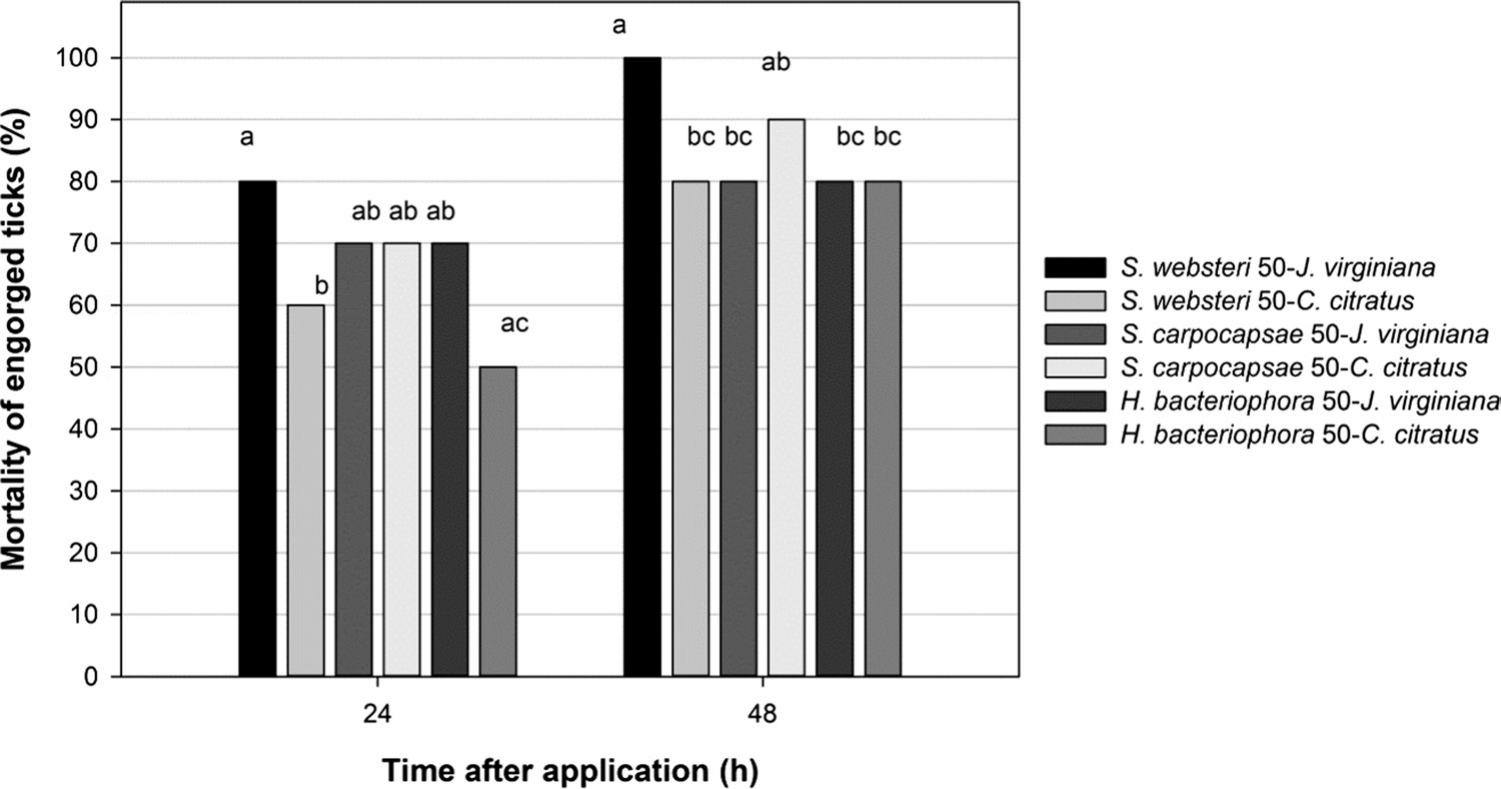
Effectivity of EPNs at concentration of 50 IJs/tick in vegetable oil emulsions at concentration of 33% on engorged adult ticks. Bars with unequal letters are statistically different (Tukey, *P* ˂ 0.05).

It can be observed that the control effectiveness (CE) of the EPNs on ticks in a period of 24 hr varied from 50 to 100%. As can be seen in Figure 3, applying the concentration of 50 IJs/tick, the treatment with the nematode *S. websteri* in *J. virginiana* oil emulsions was the one with the highest CE (80%), followed by *S. carpocapsae* also with the *J. virginiana* oil emulsion, *S. crpocapasae* in *C. citratus* emulsion, and *H. bacteriophora* in *J. virgijiana* oil emulsion. All of them had a CE higher than 70%. There were statistical differences (*P* = 0.05) in CE among the treatments mentioned above, as well as for the combinations of *S. websteri* in *C. citratus* (60%) oil emulsion, and *H. bacteriophora* in oil emulsion of *C. citratus* (50%). After 48 hr of the application, the CE increased in all the treatments. The nematode *S. websteri* in *J. virginiana* oil emulsion reached a 100% of CE; that is why it was statistically different (*P* ˂ 0.05) from the treatments *S. websteri-C. citratus*, *S. carpocapsae-J. virginiana* and *H. bacteriophora-J. virginiana* and *H. bacteriophora*–*C. citratus,* which reached 80% of CE (Fig. 3).

**Figure 4:**
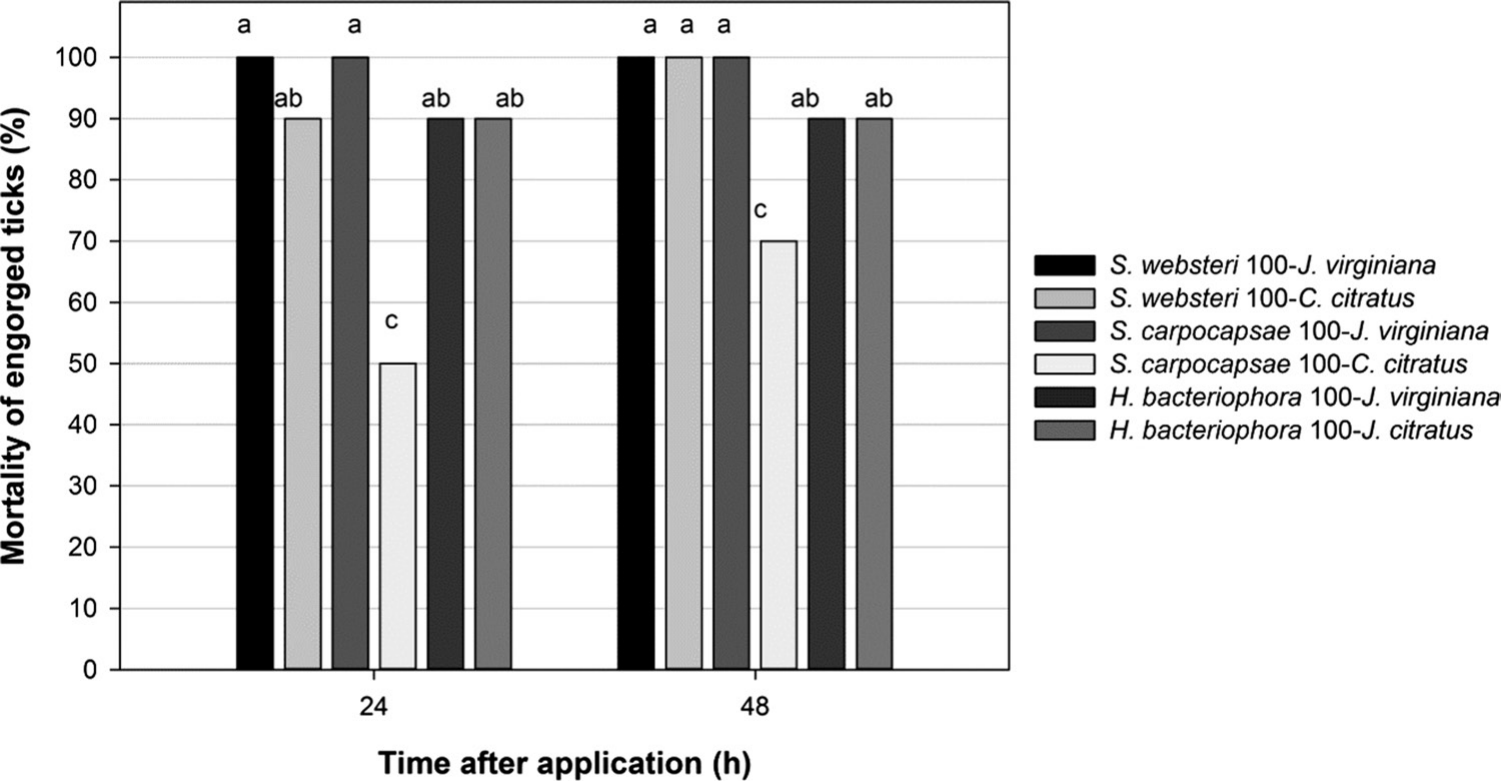
Effectivity of EPNs at concentration of 100 IJs/tick in vegetable oil emulsions at concentration of 33% on engorged adult ticks. Bars with unequal letters are statistically different (Tukey, *P* ˂ 0.05).

As can be seen in Figure 4, the concentration of 100 IJs/tick gave a CE of 100% in treatments with *S. websteri- J. virginiana* and *S. carpocapse- J. virginiana,* while three treatments showed a CE of 90% (*S. websteri-C. citratus*, *H. bacteriophora-J. virginiana* and *H. bacteriophora*-*C. citratus*). In this assay, it was found a statistical difference (*P* ˂ 0.05) among the treatments mentioned above and the treatment with *S. carpocapsae-C. citratus,* which only controlled 50% of the ticks. After 48 hr, it was found that the treatment *S. websteri-C. citratus* reached a CE of 100% on ticks, and the treatments *H. bacteriophora*–*J. v*irginiana and *H. bacteriophora*-*C. citratus* accumulated a 90% of CE. A statistical difference (*P* ˂ 0.05) was found between the treatments with *S. carpocapsae-C. citratus* and the rest of the treatments. All the combinations with a concentration of 100 IJs/tick are considered a suitable alternative for controlling these parasites. In the infectivity of ticks by EPNs verification tests, it was observed a limited emergence of IJs, which could be due to the chitinous characteristics of ticks.

### Control effectiveness on ticks in infested dogs

The treatment that produced the highest CE was *S. websteri* at 119 IJs/tick in the *J. virginiana* oil emulsion, which after 24 hr had 68% CE; after 72 hr it increased to 80%, and after 96 hr it reached 89%. The treatment with the nematode *S. websteri* at the same concentration in the *C. citratus* oil emulsion caused 57.50% mortality in a period of 24 hr and after 72 hr the solution reached 85%, being the most effective.

**Figure 5:**
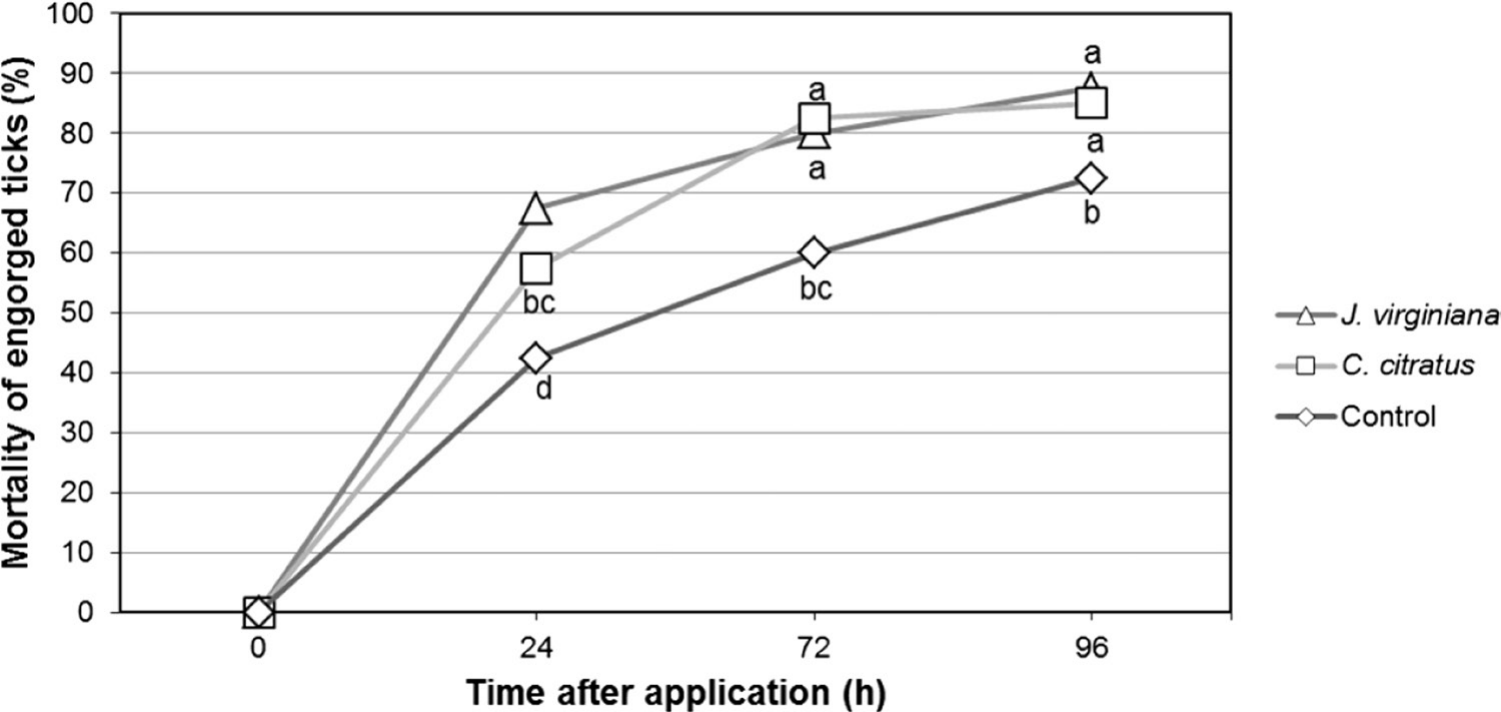
Control effectiveness of *S. websteri* at 119 IJs/tick in two vegetable oil emulsions at a concentration of 33% and a Control with *S. websteri* in only distilled water.

The control *S. websteri-*distilled water reached 42.5% of mortality on the ectoparasites in 24 hr, and increased to 60% in 48 hr. At the end of the evaluation period, the maximum CE with this treatment was 72%. This result could be due to the effect of the applied nematode (Fig. 5).

## Discussion

The survival of EPNs improved according to the essential oil used for the preparation of the emulsions. There exist just a few reported studies on the behavior of EPNs survival in vegetable oil emulsions. Alves et al. (2017) prepared an Aureo® vegetable oil emulsion containing 2,000 IJs of *Heterorhabditis* sp. (strain CB40) which increased its viability in 87.4% with regard to the control in water.

Considering that the storage temperature of the oil emulsions was stable (23 ± 3 °C) and below 35 °C, the results suggest that the IJs of *H. bacteriophora* and *S. carpocapsae* survived because they have their own biological fitness for desiccation tolerance, especially in emulsions with *C. citratus* and *J. virginiana*, both at 13% concentration.

It is known that heat and desiccation cause negative effects in the survival of the EPNs, mainly in field applications (Ruiz-Vega et al., 2011). Dito et al. (2016) establish that the survival of the nematodes is closely linked to their metabolism, where temperature is a determinant variable. Temperature increases the mobility of the IJs and their lipids expenditure, which causes starvation and physical damage and the increment of mortality (Cagnolo and Campos, 2008).

Tolerance to heat and desiccation in the nematodes *S. carpocapsae* and *H. bateriophora* is often reported, but there is variability among strains of the same species; however, for *S. websteri,* there are no published studies about their tolerance to heat and desiccation. Ruiz-Vega et al. (2011) reported a 70% survival of *H. bacteriophora* at 35 °C, while in a range of water activity (A_w_) of 0.86 to 0.97 its survival went from 23.6 to 59.3%. This affirmation was confirmed by Ulu and Susurluk (2014), as they found the same tendency in relation to heat and desiccation in 10 strains of *H. bacteriophora* isolated from different climatic regions in Turkey.

In this study it was observed that from the lowest concentration of IJs used, which was of 50 IJs/tick and that caused 66.66% of mortality, the CE increased when the concentration of IJs was higher. There are no registers of studies of *S. websteri* against these ectoparasites, but it was the most effective nematode against the engorged adult ticks. The LC_50_ and the LC_90_ estimated were 32.2 IJs/tick and 119 IJs/tick, respectively. These are the lowest when compared with Freitas-Ribeiro et al. (2005) who estimated the LD_50_ and the LD_90_ of *S. carpocapsae* (all strain) for a period of CE of 3 d in 156 IJs and 5,001 IJs, respectively.

The EPNs are effective agents for biological control of engorged adult ticks. Some CE reports in laboratory with *H. bacteriophora* and *S. carpocapsae* have been published, but there are not any reported results on the CE of the *S. websteri* nematode on adult or nymphs ticks. Regarding *S. carpocapsae,* it was considered a moderately effective nematode for control of adult ticks and similar results have been found by other authors. Zhioua et al. (1995) found that *S. carpocapsae* was a pathogen for the full female ticks (*Ixodes scapularis* Say), although nothing was said about the concentrations of the nematode used. In laboratory trials at 25 ± 1 °C, Butt et al. (2016) found that *S. carpocapsae* (Turkish strain) at a concentration of 4,000 IJs was slightly more infectious against engorged adults (30–40%) than against nymphs (36%), but it required an exposure period of 14 d to the IJs; however, there was not any emergence of EPNs from the infected cadavers. The exposition of *Boophilus microplus* during 72 hr to the concentration of 6,000 IJs was more effective against male and female engorded adults (>90%) after 7 to 6 d under lab conditions at 27 ± 1 °C and 80% relative humidity (Freitas-Ribeiro et al., 2005).

The percentage of control of adult female engorged ticks of *Rhipicephalus microplus* exposed to the nematode *H. bacteriophora* (HP88) in a concentration of 300 IJs/tick was of 95.6% (Monteiro et al., 2013). The authors applied the nematodes *H. bacteriophora* (HP88) and *H. indica* (LPP1) in concentration of 300 IJs combined with *Lippia sidoides* essential oil on engorged adult female ticks of *Rhipicephalus microplus* at 27 ± 1 °C and a relative humidity of 80 ± 10%, which controlled effectively the ectoparasites (>99%).

In the laboratory, Monteiro et al. (2013) found that a concentration of 300 IJs/female of *H. bacteriophora* (HP88) was enough to cancel 100% of the oviposition of the non-parasitic phase of *R. microplus* with a time of exposure of 48 hr, which suggests that the longer times of exposure permit the nematode to localize and penetrate the host.

The application of EPNs in insect cadaver formulations in laboratory conditions (27 ± 1 °C and RH of 80 ± 10%) on engorged *R. microplus* females was tested by Monteiro et al. (2014) and found a higher cumulative mortality using *H. bacteriophora* HP88 and *S. carpocapsae* all, with 100 and 80.6% CE, respectively. Using *H. bacteriophora* Poinar (VS strain) and *S. carpocapsae* Weiser (All strain) against *R. microplus* (Deutch strain), the maximum mortality reported by Singh et al. (2018a) was 15 and 20%, respectively. In bioassays in petri dishes containing a 1.0-ml distilled water suspension at concentrations of 1,250, 2,500, and 5,000 IJs per dish, the maximum mortality of *R. microplus* ticks was 85% at the largest concentration of *S. carpocapsae* Weiser (All strain) and 45% with the largest concentrations of *H. bacteriophora* (Singh et al., 2018b).

In our study, the application of EPNs in oil and water emulsions on the domestic dogs’ fur increased the CE on the adult ticks. Even though vegetable oils and EPNs have been used for the control of adult ticks in laboratory (Samish and Glazer, 2001; Ellse and Wall, 2014), the studies about their combined application in the field on domestic dogs are limited.

The action of some vegetable oils against ticks is through repellency (Benelli and Pavela, 2017), while the action of the EPNs is by pathogenicity and virulence (Samish and Glazer, 2001). The concentration of citronela oil (*Corymbia citriodora* (Hook.) at 1% applied against *Dermacentor reticulatus* Fab. was able to repel 76.6 to 86.9% of ticks. Against *Amblyoma americanum* L., the concentration of *Juniperus communis* L. and *Juniperus chinensis* L. oils were able to repel 50% of the ectoparasites in 15 minutes.

As it was showed in the results obtained in this study, the contact time with the insect-pest is a determinant factor to increase the CE of the EPNs, which agrees with Monteiro et al. (2012) and Hill (1998). The last author explains that the exposure time of the ticks to the EPNs for a short period reduces the number of IJs that successfully penetrate in the engorged female ticks reducing the mortality rate of the host.

## Conclusions

The combined application of vegetable oil and EPNs could be a good alternative for the control of the ticks, as it was observed that the time of contact and the kind of oil are the determinant factors to increase CE.

The oil emulsion of *C. citratus* and *J. virginiana* at 13% favored the survival of the nematodes (*S. carpocapsae, H. bacteriophora,* and *S. websteri*) in 55 and 60% for a period of 96 hr, respectively.

In lab applications, the combination of the nematode *S. websteri* (in doses of 50 and 100 IJs) in oil emulsions of *J. virginiana* at 33% concentration was highly lethal on engorged adult ticks in the first 24 hr after the application, with a CE of 80 to 100%.

In field applications on domestic dogs, it was found that the DL_90_ of 119 IJs of *S. websteri* in oil emulsions of *J. virginiana* (33%) had the best performance with an accumulated CE of 89%, 96 hr after being applied.

The results suggest that the treatment with the nematode *S. websteri* in oil emulsions of *J. virginiana* or *C. citratus* at a concentration of 100 IJs per tick has the right potential to obtain a good percentage of control in dogs with presence of ticks, because it only required from 24 to 48 hr to be effective.
